# The mediating role of social support in promoting physical activity among children in South Africa

**DOI:** 10.4102/sajp.v79i1.1896

**Published:** 2023-10-25

**Authors:** Howard Gomwe, Lesego Phiri, Chioneso Show Marange

**Affiliations:** 1 Skills Centre, Faculty of Health Sciences, Sefako Makgatho Health Sciences University, Pretoria, South Africa; 2 Department of Statistics, Faculty of Science, University of Fort Hare, East London, South Africa

**Keywords:** family encouragement, teacher encouragement, peer encouragement, physical activity, children

## Abstract

**Background:**

Low physical activity (PA) participation levels and increasing non-communicable diseases in children are concerning in South Africa and globally.

**Objectives:**

We sought to assess the mediating role of perceived social support factors on the relationship between PA enjoyment and PA levels among rural, peri-urban and urban school children.

**Method:**

A cross-sectional study was adopted to assess peer, family and teacher encouragement as mediators on the relationship between perceived PA enjoyment and perceived physical activity participation among children, using the Physical Activity Questionnaire for Older Children (PAQ-C). The sample comprised a random sample of primary school learners aged 9–14 years.

**Results:**

The sample consisted of 870 primary school learners with a mean age of 11.0 ± 1.49 years. Most participants were girls (*n* = 519; 59.7%). The findings suggest low levels of perceived PA participation (mean = 2.33, standard deviation [s.d.]: 0.43). Peer (β = 0.0187, 95% confidence interval [CI]: 0.0088, 0.0307), family (β = 0.0280, 95% CI: 0.0155, 0.0425) and teacher (β = 0.0242, 95% CI: 0.0127, 0.0378) encouragement partially mediates the relationship between perceived PA enjoyment and perceived PA participation. Family encouragement (β = 0.0158, 95% CI: 0.0017, 0.0311) has the most considerable mediating effect, followed by teacher encouragement (β = 0.0125, 95% CI: 0.0010, 0.0269).

**Conclusion:**

The findings demonstrated low levels of perceived PA participation in school learners. Therefore, we recommends including social factors as mediators in PA intervention programmes in primary schools.

**Clinical implications:**

Social support factors as mediators on the relationship between PA enjoyment and PA participation among children may improve children’s PA participation levels and help prevent non-communicable diseases in future.

## Introduction

Physical activity (PA) involves the most minor to most complex planned and unplanned movements made by individuals, which results in energy consumption (Coetzee, Du Toit & De Vos 2016). Physical activity can also be regarded as daily routine activities like running, walking and moderate to vigorous body movements (eds. Kohl & Cook 2013). Participation in physical activity helps to prevent chronic diseases like hypertension, diabetes, obesity, cardiovascular diseases, cancer and depression in children, while physical inactivity is detrimental to their health (Menescardi & Estevan 2021). However, most children between 5 and 17 years old need to meet the recommended participation in PA of at least 60 min from moderate to vigorous PA per day (Bull et al. 2020). A low level of PA participation is usually due to certain obstacles in children’s environment (Estevan et al. 2021).

The use of behavioural theory is important when designing PA interventions as the theoretical constructs can help researchers determine how the intervention works and how future interventions can be modified and improved (Michie & Abraham 2004). In our study, the social cognitive theory (Bandura 1986; Bandura & Walters 1997) gives a good theoretical basis for assessing the behavioural, social and motivational factors associated with PA behaviours. The social cognitive theory talks about how children behave through interaction with peers, siblings, teachers and parents at home and school. The theory has been grounded as a basis for understanding PA behaviours. According to Bandura (1986), school children learn certain behaviours from others but sometimes acquire behaviour themselves. The social cognitive theory is made up of three components, namely social support, self-efficacy and enjoyment.

Research on the effect of social support factors on PA among children is highly recommended in public health (Cheng et al. 2020). The differences in PA between boys and girls may be caused by differences in social support provided by parents and peers (Cheng et al. 2020). In addition, schools are regarded as ideal places to promote PA participation levels in children, as school-based interventions have proved to be effective (Eather, Morgan & Lubans 2013). Assessing the mediating role of perceived social support factors on the relationship between PA enjoyment and PA participation levels among children can help design interventions to increase PA participation levels and help prevent chronic diseases. Studies have also been encouraged to explore and report mediators of PA change in children’s interventions (Lubans, Foster & Biddle 2008).

Social support has a significant influence in promoting school children’s PA participation levels (Biddle & Goudas 1996; Davison 2004; Sallis, Prochaska & Taylor 2000). Social support is defined as school children’s support and help from family, peers and acquaintances. It is also viewed as any form of support given by guardians, parents and peers that assist school children in increasing PA behaviour (Monteiro, Rodrigues & Lopes 2021). It has been shown in many studies that social support has many benefits. The studies have shown that parental, teacher and peer support are associated with improved PA participation among children and adolescents (Haidar et al. 2019; Korcz et al. 2018). Social support has been shown to be associated with children’s engagement in regular PA participation. Studies show that social support brings self-efficacy in children’s moderate and vigorous PA participation levels (Biddle & Goudas 1996; Johnson et al. 2000). In a study by Van Der Horst et al. (2007), results indicated that the age of the children strongly influences PA participation level.

Family encouragement describes any form of support from parents that enables the children to go and participate in sporting activities. These include activities such as buying equipment or paying fees and transportation (Duncan, Duncan & Strycker 2005; Prochaska, Rodgers & Sallis 2002). Engaging in these activities promotes lifestyle behaviour (Biddle & Goudas 1996; Dowda et al. 2007). Some families have greater encouragement, which helps shape important attitudes and behaviours associated with PA participation (Edwardson et al. 2013). This means that families can act as role models in their homes to increase PA participation in children. Evidence shows that parental support of school learners in PA participation also contributes to maintaining PA participation habits (Tremblay et al. 2015). It is important to note that a lack of family and friend encouragement decreases PA participation at the adolescent stage (Campos et al. 2019).

On the other hand, peers also play a pivotal role in influencing the behaviour of other peers (Smith 1999). During adolescence, peers share emotional support and friendship. Peers like to behave the same to fit into the company of others. This type of friendship is common during childhood and adolescence (Smith 1999). Physical activity participation is an important common ground for peer interaction, and it provides a conducive environment for learning the rules of the games they enjoy playing, and by so doing, it increases PA participation levels. A study by Smith (1999) showed that a higher understanding of peer acceptance and perceptions of good friendships positively affected PA participation. As children grow up, they distance themselves from their parents and spend more time with peers or hanging around with friends. Peer support within the communities can help with the creation of clubs in the community where learners live. These clubs would then create teams for sporting activities in the communities and then participate in PA with peer involvement (Beets, Pitetti & Forlaw 2007). The support from parents and peers has the same impact, but peer support exceeded parental support only for ages between 16 and 18 years (Bokhorst, Sumter & Westenberg 2010). Research suggests that peers have more influence on adolescent development in PA participation (Van Der Horst et al. 2007).

The school teacher has a role in the children’s PA participation. Children spend most of their time at school and are more influenced by teachers and classmates, and this is a notable adolescent development (WHO 2010). Teachers impart knowledge and skills through the sporting games played by children. The teacher’s influence starts in the classroom when teaching life orientation (or physical education) and extra-curricular sports in the school. The learners will then apply the skills taught in class and the extra-curricular activities to participate in physical activities to improve their healthy lifestyle throughout their lives (Fairclough, Stratton & Baldwin 2002; Sallis et al. 1997). Thus, physical education classes at school build intrinsic motivation in children to live a healthy lifestyle (Bortoli et al. 2018; Vitali et al. 2019). However, Bokhorst et al. (2010) revealed that teacher support is lower in older age groups.

The differences in gender are important considerations in understanding the impact of social support from peers, parents, teachers and its PA participation levels relationship (Edwardson et al. 2013). Boys and girls have different choices for social support. These differences may influence the relationship between PA participation and different sources of social support (Edwardson et al. 2013). Studies have also shown that boys tend to receive more peer support to engage in PA than girls, with girls often receiving little to no peer support (Edwardson et al. 2013). In a study by Bronikowski et al. (2018), the results showed that boys get more support from teachers, while on the other hand, Edwardson et al. (2013) reported that girls have greater social support in general. Bokhorst et al. (2010) also reported that girls receive more support from teachers, peers and classmates than boys. The residential area also influences the level of PA participation. Rural children participate more in PA than urban and peri-urban children (Gomwe et al. 2022). This may be attributed to the lifestyle differences within the environmental settings. In rural areas, most learners walk long distances and engage in more physical household chores than in urban and peri-urban settings (Gomwe et al. 2022). Exploring mediators of PA participation in children can help to modify effective behaviour change interventions (Salmon, Brown & Hume 2009; Lubans et al. 2008). Our study seeks to assess the mediating role of perceived social support factors on the relationship between physical activity enjoyment and physical activity participation levels among rural, peri-urban and urban school children.

## Methods

### Study design and participants

A cross-sectional descriptive study was used for data collection in which social support, self-efficacy and physical activity enjoyment were measured among the children. A total of 876 schoolchildren aged 9–14 years participated in our study. Primary school learners aged between 9 years and 14 years who were not physically disabled were included.

**FIGURE 1:**
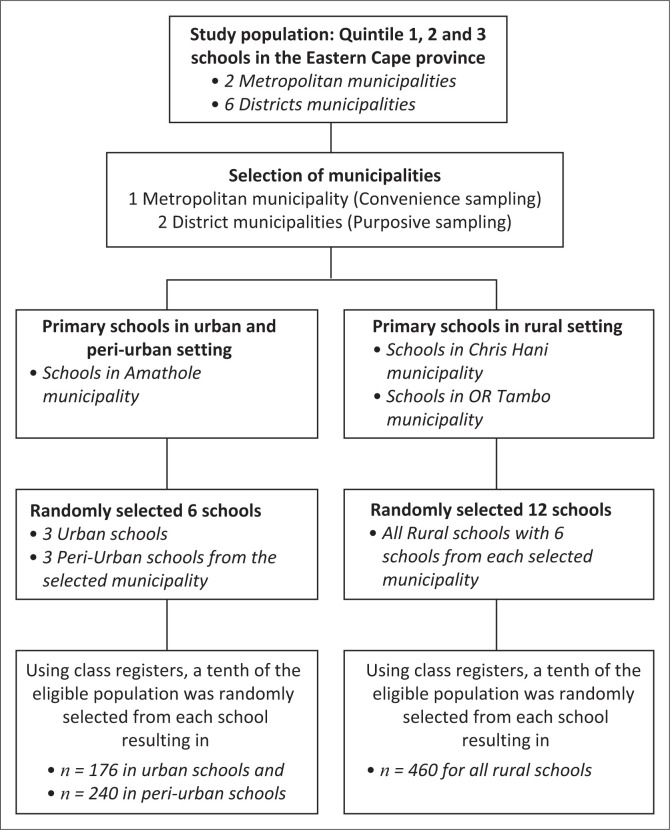
Flowchart for sampling and sample determination.

A total of 18 primary schools were selected at random. Thus, schools were first arranged and numbered alphabetically using a database of all primary schools (obtained from the Department of Education in the Eastern Cape province) in the metro and the two district municipalities. Schools were clustered according to the respective metro and district municipalities. Each cluster generated six random numbers, resulting in 18 primary schools being selected for our study. Using a tenth of the eligible population as a sample size was an arbitrary choice based on the available resources, time and logistical constraints. We chose this approach with complete confidence that the resultant sample would still achieve the required sample size and provide meaningful insights while being manageable within the study limitations. In each randomly selected school, Grades 5–7 were purposively chosen. The teachers were asked to provide the class registers, and in each class, learners were numbered in alphabetical order of their surnames. A pre-determined sample was established for each class, 10% of the total learners. The sample was selected by choosing even numbers from the numbered class register list. Once the predetermined number was reached, the chosen learners were given a consent form to be signed by a parent or guardian. This was repeated for each class (Grades 5–7) for each school to have a tenth of the eligible population randomly selected from each school.

### Measures

#### Perceived physical activity participation

The Physical Activity Questionnaire for Older Children (PAQ-C), which has been previously used and validated in an ethnically diverse and similar cohort of South African children, was used (McVeigh & Norris 2012; Van Biljon et al. 2018). It measures general PA levels in children aged 8–14 years during a typical week in a school year. The questionnaire gathered information about the child’s participation in various physical activities across several domains. These domains included school physical education, informal activities, sedentary activities after school, transport to and from school and extramural activities. The questionnaire was piloted on a convenient sample of children from the same age group and similar demographic profiles prior to the main data collection. This sample did not form part of the final analysis. The primary purpose of the pilot testing was to ensure clarity of the terminology used and the applicability and availability of the listed sports activities. Modifications to certain parts of the questionnaire were performed after the pilot study; for example, cross-country skiing, ice hockey and badminton were removed and replaced with soccer, athletics and rugby to suit the local context. A final mean score categorised learners as having low, moderate or high PA levels. Low levels of PA were 1.00 to 2.33, moderate levels 2.34 to 3.66 and high levels 3.67 to 5.00 (Kowalski, Crocker & Donen 2004). The PAQ-C has demonstrated good internal consistency (Moore et al. 2007) and acceptable test-retest reliabilities (Crocker et al. 1997). Convergent and construct validity of the PAQ-C has also been established, with moderate associations between the activity rating scale (Kowalski et al. 1997). Our study reported internal consistency of α = 0.62, a statistically acceptable reliability score.

#### Perceived Physical Activity Enjoyment Scale

We adopted the Physical Activity Enjoyment Scale (PACES), which is an 18-item tool used to measure perceived physical activity enjoyment (Crocker, Bouffard & Gessaroli 1995; Kendzierski & DeCarlo 1991). Participants were asked to rate various physical activity enjoyment items using a 5-point Likert scale (1 – Strongly Disagree, 2 – Disagree, 3 – Neutral, 4 – Agree and 5 – Strongly agree) with higher PACES scores reflecting greater levels of perceived physical activity enjoyment. A score for enjoyment was computed by calculating the average of the 18 items. The tool has demonstrated internal consistency in several studies, including in 12–16-year-old children, with a coefficient α = 0.90 (Crocker et al. 1995). The 18-item PACES was found to be internally reliable in our sample (α = 0.66).

#### Social support factors

The social environment support was measured through the frequency of family and siblings and peer and teacher support for involvement in physical activities. We focused on the peer, family and teacher support factors. These social support factors have been measured and used in various studies (e.g., see Birnbaum et al. 2005; McMinn et al. 2008; Morrissey et al. 2015; Prochaska et al. 2002; Saunders et al. 1997, among others). We adopted the social support factors scales from Prochaska et al. (2002) and Morrissey et al. (2015). Learners were asked to indicate how often their family members or siblings at home, friends or peers and teachers at school encourage them to participate in physical activities during their leisure time or do physical activity with them, provide transportation to do physical exercise and praise them in physical activity or watch them during physical activities. For example, on a 5-point Likert scale, the family and teacher questions asked learners to consider ‘the last 7 days’ and respond to: … how frequently family members or teacher/s encouraged you to do physical activity? On peer support, we asked the participants to think about ‘the last 7 days’ and respond to: … how frequently did you encourage your friends to do physical activity or play sports?; … how frequently did your friends encourage you to do physical activity or play sports? The peer item on ‘how frequently did you encourage your friends to do physical activity or play sports?’ was not included in the analysis as it asks participants how they provide support to their peers rather than how they perceive their peers to provide support to them. These support factors have been widely used, and their reliability and validity have been widely reported (Prochaska et al. 2002). In our study, the Cronbach’s alpha for these scales indicated an internal consistency of 0.64 for the family support scale, 0.67 for the peer support scale and 0.69 for the teacher support scale.

### Hypotheses and conceptual framework

The following null hypotheses were formulated:

**Hypothesis 1:** Peer encouragement mediates the relationship between physical activity enjoyment and perceived physical activity level among primary school children.

**Hypothesis 2:** Family encouragement mediates the relationship between physical activity enjoyment and perceived physical activity level among primary school children.

**Hypothesis 3:** Teacher encouragement mediates the relationship between physical activity enjoyment and perceived physical activity level among primary school children.

**FIGURE 2:**
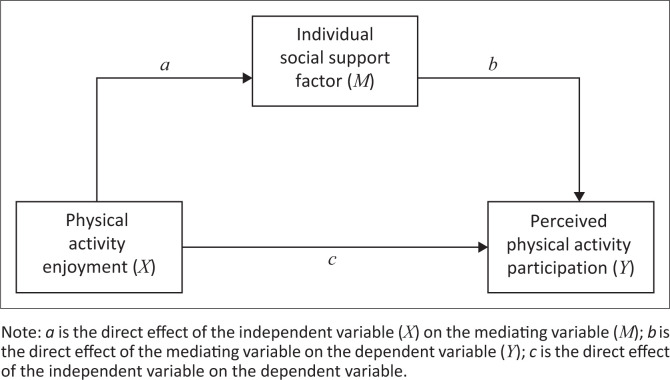
Simple multi-level mediation conceptual model for the mediating effect of individual social support factors on the relationship between physical activity enjoyment and perceived physical activity participation.

We proposed a conceptual model for our study from the hypothesised frameworks. The conceptual model is presented in Figure 2. The model represents the simple multi-level mediation conceptual model for the mediating effect of the individual social support factors on the relationship between physical activity enjoyment and perceived physical activity participation.

### Data analysis

The Statistical Package for the Social Sciences (SPSS) version 27 and the MLmed macro for SPSS (Hayes & Rockwood 2020) were used for data analysis. A descriptive analysis was then used to describe the demographic features and theoretical variables. The Cronbach’s alpha coefficient was used to assess the reliability of the theoretical variables. The non-parametric Mann–Whitney *U* and Kruskal–Wallis tests were used to compare the various theoretical variables on the categorical demographic features. Because of clustering, a multi-level mediation analysis was adopted. The MLmed macro was then used to address the hypothesised frameworks. Following MacKinnon, Fairchild and Fritz (2007), the strength of the indirect and the direct effects were used to determine the result of the multi-level mediation analysis. As we focused on three mediator variables, which are well-known social support factors, it was also interesting to know if they drive the mediation more than the others. A post-hoc analysis was conducted using parallel multi-level mediation.

### Ethical considerations

Ethical clearance to conduct the study was obtained from the University of Fort Hare Ethics Committee (LY0011SGOM01). We obtained permission letters from the Department of Health, Department of Education and school principals from the participating schools. As the participants were minors, parental or guardian consent was obtained, and participation was voluntary.

## Results

### Descriptive analysis

Out of 900 potential participants, 876 participated, giving a response rate of 97.3%. Of these, 870 usable responses were used for the data analysis. Table 1 shows the demographic characteristics of the selected participants. The mean age was 11.0 ± 1.49 years, and most participants were girls (*n* = 519; 59.7%). Most of these children resided in rural areas (*n* = 459; 52.8%), while 235 (27.0%) lived in peri-urban areas. Of the 870 children, 76 (20.2%) were from urban-based primary schools.

**TABLE 1:** Demographic characteristics of participants.

Characteristic	*n*	Valid %	Mean ± s.d.
**Gender**			
Boys	351	40.3	-
Girls	519	59.7	-
**Age (years)**	-	-	11.0 ± 1.49
**Age categories**			
9 to 10 years	222	25.5	-
11 to 12 years	423	48.6	-
13 to 14 years	225	25.9	-
**Residence**			
Urban	176	20.2	-
Peri-urban	235	27.0	-
Rural	459	52.8	-

s.d., standard deviation.

Table 2 shows the mean levels summary with respective non-parametric mean comparisons for perceived physical activity participation, perceived physical activity enjoyment and social support factors by gender, age and residence. Measured on a 5-point Likert scale, most variables reported moderate mean levels except for perceived physical activity participation. Thus, perceived physical activity participation (mean = 2.33, standard deviation [s.d.]: 0.43) had a low mean rating, suggesting low levels of perceived physical activity participation by primary school children.

**TABLE 2a:** Mean rank summary with respective non-parametric comparisons.

Gender	Combined (*n* = 870)	Boys (*n* = 351)	Girls (*n* = 519)	*p*
**Theoretical variables’ mean rank summary by gender (Mean of combined groups are also presented)**				
Perceived PA participation	2.33 ± 0.43	466.60	414.47	0.003*
Perceived PA enjoyment	3.32 ± 0.54	417.86	445.74	0.108
Peer encouragement	3.07 ± 0.76	436.65	434.72	0.911
Family encouragement	3.19 ± 0.92	420.51	445.64	0.147
Teacher encouragement	3.16 ± 0.91	423.27	443.77	0.237

PA, physical activity.

* , Statistically significant differences at *p* < 0.05.

**TABLE 2b:** Mean rank summary with respective non-parametric comparisons.

Age	9–10 years (*n* = 222)	11–12 years (*n* = 423)	13–14 years (*n* = 225)	*p*
**Theoretical variables’ mean rank summary by age**				
Perceived PA participation	421.7‡	420.3‡	477.6†	0.014*
Perceived PA enjoyment	447.2	443.2	405.6	0.130
Peer encouragement	409.9	450.2	433.2	0.148
Family encouragement	407.7‡	466.5†	404.7‡	0.002*
Teacher encouragement	402.1	447.7	445.0	0.071

PA, physical activity.

* , Statistically significant differences at *p* < 0.05.

†, ‡ , Grouping for the Kruskal–Wallis post-hoc pairwise comparisons, and represents statistically significant different mean levels using asymptotic significances (2-sided tests) adjusted by the Bonferroni correction for multiple tests.

**TABLE 2c:** Mean rank summary with respective non-parametric comparisons.

Residence	Urban (*n* = 176)	Peri-urban (*n* = 235)	Rural (*n* = 459)	*p*
**Theoretical variables’ mean rank summary by residence**				
Perceived PA participation	316.7‡	312.5‡	544.0†	< 0.0001*
Perceived PA enjoyment	436.0	406.5	448.3	0.115
Peer encouragement	468.1	424.9	428.4	0.150
Family encouragement	481.2†	448.1‡	411.5‡	0.005*
Teacher encouragement	415.0‡	412.4‡	455.2†	0.048*

PA, physical activity.

* , Statistically significant differences at *p* < 0.05.

†, ‡ , Grouping for the Kruskal–Wallis post-hoc pairwise comparisons, and represents statistically significant different mean levels using asymptotic significances (2-sided tests) adjusted by the Bonferroni correction for multiple tests.

The mean levels of PA in boys (mean = 2.39; s.d.: 0.44; *n* = 351) are considered moderate, while that of girls (mean = 2.29; s.d.: 0.42; *n* = 351) are considered low. According to the Mann–Whitney *U* test, the levels of perceived physical activity participation were significantly higher (*p* = 0.003) in boys (mean rank = 466.60) as compared to that of girls (mean rank = 414.47). There were no statistically significant differences in the levels of other theoretical variables by gender.

Regarding mean comparisons by age, the Kruskal–Wallis *H* test revealed a statistically significant difference (*p* = 0.014) in perceived physical activity participation levels for the different age groups. Using the Kruskal–Wallis post-hoc pairwise comparisons, the 13–14 year age group (mean rank = 477.6) had a significantly higher perceived physical activity participation level than the other age groups. Statistically significant differences were also observed in the ratings of family encouragement across the different age groups (*p* = 0.002). The Kruskal–Wallis post-hoc pairwise comparisons revealed that the 11–12 year age group (mean rank = 466.5) had a significantly higher rating for perceived family encouragement than the other age groups.

Table 2 presents the comparisons for perceived physical activity participation, perceived physical activity enjoyment and social support factors by residence. The Kruskal–Wallis *H* test revealed statistically significant differences in perceived physical activity participation levels by residence (*p* ≤ 0.0001). The Kruskal–Wallis post-hoc pairwise comparisons showed that the levels of perceived physical activity participation of rural learners (mean rank = 544.0) were significantly higher than that of peri-urban (mean rank = 312.5) and urban learners (mean rank = 316.7). Statistically significant differences were also reported in the ratings of family encouragement (*p* = 0.005) and teacher encouragement (*p* = 0.048) across the different age groups. The Kruskal–Wallis post-hoc pairwise comparisons showed that the ratings for perceived family encouragement were significantly higher for urban-based learners (mean rank = 481.2) followed by those in peri-urban settings (mean rank = 22.1) and lastly, rural-based learners (mean rank = 411.5). On the contrary, learners from rural areas (mean rank = 455.2) reported significantly higher ratings on perceived teacher encouragement than those from urban and peri-urban areas.

### Simple mediation analysis

After establishing that the data meets the multiple regression assumptions, the authors conducted the mediation analysis. A simple multi-level mediation was undertaken to determine the mediating effect of peer encouragement on the relationship between perceived physical activity enjoyment and participation. We opted for 20 000 bootstrap samples and utilised the bias-corrected bootstrapping method for the mediation analysis. The findings for the simple multi-level mediation analysis are presented in Table 3. Results indicated that perceived physical activity enjoyment had a significant direct and positive effect on the mediator variable peer encouragement (β = 0.2689, *t* = 5.5819, *p ≤* 0.0001). In addition, peer encouragement had a significant direct and positive effect on perceived physical activity participation (β = 0.0696, *t* = 4.2035, *p* ≤ 0.0001). While controlling the mediator variable (i.e., peer encouragement), the results of the multi-level mediation analysis indicated that perceived physical activity enjoyment was a significant predictor of perceived physical activity participation (β = 0.0997, *t* = 4.2040, *p* ≤ 0.0001). The indirect effect was statistically significant (β = 0.0187, 95% confidence interval [CI]: 0.0088, 0.0307). Thus, peer encouragement partially mediates the relationship between perceived physical activity enjoyment and perceived physical activity participation.

**TABLE 3:** Simple multi-level mediation analysis to determine the mediating effect of peer, family and teacher encouragement on the relationship between perceived physical activity enjoyment and perceived physical activity participation.

Effects	Unstandardised beta coefficients		Significance of beta coefficients			95% confidence interval	
	Beta	s.e.	*t*-value	*z*-value	*p*	LLCI	ULCI
**Peer encouragement as a mediator variable**							
Fixed effects (within-effects specified) - Specific direct effect(s)							
*PAE (X) → PE (M*_1_*)*	0.2689†	0.048	5.5819	-	< 0.0001	0.1744	0.3635
*PE (M*_1_*) → PAP (Y)*	0.0696†	0.016	4.2035	-	< 0.0001	0.0371	0.1021
*PAE (X) → PAP (Y)*	0.0997†	0.023	4.2040	-	< 0.0001	0.0532	0.1463
Within – Indirect effect(s)							
*PAE → PE → PAP*	0.0187†	0.005	-	3.3243	0.0009	0.0088	0.0307
**Family encouragement as a mediator variable**							
Fixed effects (within-effects specified) - Specific direct effect(s)							
*PAE (X) → FE (M*_2_*)*	0.4009†	0.0562	7.1167	-	< 0.0001	0.2903	0.5114
*FE (M*_2_*) → PAP (Y)*	0.0699†	0.0142	4.4555	-	< 0.0001	0.0422	0.0976
*PAE (X) → PAP (Y)*	0.0904†	0.0238	3.7853	-	0.0002	0.0435	0.1373
Within – Indirect effect(s)							
*PAE → FE → PAP*	0.0280†	0.0064	-	4.0400	0.0001	0.0155	0.0425
**Teacher encouragement as a mediator variable**							
Fixed effects (within-effects specified) - Specific direct effect(s)							
*PAE (X) → TE (M*_3_*)*	0.3652†	0.0562	6.5016	-	< 0.0001	0.2549	0.4754
*TE (M*_3_*) → PAP (Y)*	0.0662†	0.0142	4.6751	-	< 0.0001	0.0384	0.0940
*PAE (X) → PAP (Y)*	0.0943†	0.0238	3.9584	-	0.0001	0.0475	0.1410
Within – Indirect effect(s)							
*PAE → TE → PAP*	0.0242†	0.0064	-	3.7664	0.0002	0.0127	0.0378

s.e., standard error; *X*, predictor/independent variable; *Y*, outcome/dependent variable; *PAE*, physical activity enjoyment; *PAP*, physical activity participation; *PE*, peer encouragement; *FE*, family encouragement; *TE*, teacher encouragement; *M*_1_, mediator variable 1; *M*_2_, mediator variable 2; *M*_3_, mediator variable 3.

† , Significant effect at alpha = 0.05.

A simple multi-level mediation analysis was also conducted to investigate the hypothesis that family encouragement mediates the relationship between perceived physical activity enjoyment and perceived physical activity participation. The findings from Table 3 show that perceived physical activity enjoyment had a significant direct and positive effect on the mediator variable family encouragement (β = 0.4009, *t* = 7.1167, *p* ≤ 0.0001). The results also revealed that family encouragement had a significant direct and positive effect on perceived physical activity participation (β = 0.0699, *t* = 4.4555; *p* ≤ 0.0001). Controlling for the mediator variable, perceived physical activity enjoyment was a significant predictor of perceived physical activity participation (β = 0.0904, *t* = 3.7853, *p* = 0.0002). A 95% bias-corrected confidence interval based on 20 000 bootstrap samples indicated that the indirect effect (β = 0.0280) was statistically significant (95% CI: 0.0155, 0.0425). In conclusion, family encouragement partially mediates the relationship between perceived physical activity enjoyment and perceived physical activity participation.

A simple multi-level mediation analysis was also conducted to investigate the hypothesis that teacher encouragement mediates the relationship between perceived physical activity enjoyment and perceived physical activity participation (see Table 3). Perceived physical activity enjoyment had a significant direct and positive effect on the mediator variable (β = 0.3652, *t* = 6.5016, *p* ≤ 0.0001). The results also revealed that teacher encouragement had a significant direct and positive effect on perceived physical activity participation (β = 0.0662, *t* = 4.6751, *p* ≤ 0.0001). While controlling for teacher encouragement, the results indicate that perceived physical activity enjoyment significantly predicted perceived physical activity participation (β = 0.0943, *t* = 3.9584, *p* = 0.0001). The results further showed that the indirect effect was statistically significant (β = 0.0242, 95% CI: 0.0127, 0.0378). These findings are consistent with partial mediation. Thus, teacher encouragement partially mediates the relationship between perceived physical activity enjoyment and perceived physical activity participation.

### Post-hoc analysis using parallel multi-level mediation

**FIGURE 3:**
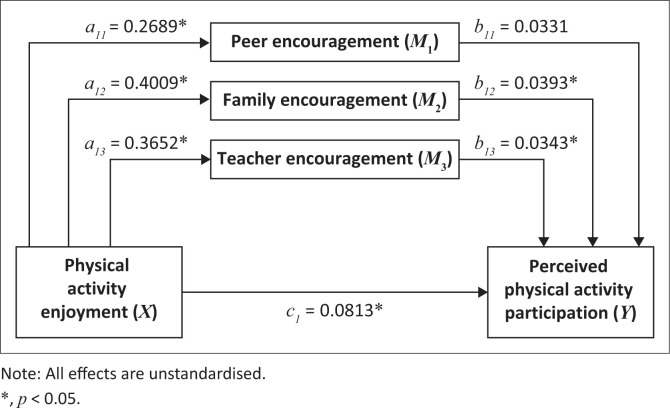
Parallel multi-level mediation model for the mediating effect of social support factors on the relationship between perceived physical activity enjoyment and perceived physical activity participation.

The results of our simple mediation suggest that all the social support factors partially mediate the relationship between perceived physical activity enjoyment and perceived physical activity participation. It would be interesting to know if these dimensions drive the mediation more than the others. To address this, we adopted a parallel multi-level mediation analysis. It can be seen in Table 4 and Figure 3 that perceived physical activity enjoyment had a significant, direct and positive effect on peer encouragement (β = 0.2689, *t* = 5.5819, *p* ≤ 0.0001), family encouragement (β = 0.4009, *t* = 7.1167, *p* ≤ 0.0001) and teacher encouragement (β = 0.3652, *t* = 6.5016, *p* ≤ 0.0001). However, peer encouragement (β = 0.0331, *t* = 1.7602, *p* = 0.0787) had no significant direct effect on perceived physical activity participation. While controlling for the mediators, the results indicate that perceived physical activity enjoyment significantly predicted perceived physical activity participation (β = 0.0813, *t* = 3.3885, *p* = 0.0007). The indirect effects showed that only the indirect effects for family encouragement (β = 0.0158, 95% CI: 0.0017, 0.0311) and teacher encouragement (β = 0.0125, 95% CI: 0.0010, 0.0269) were statistically significant. Thus, family encouragement drives the mediation between perceived physical activity enjoyment and perceived physical activity participation followed by teacher encouragement.

**TABLE 4:** Parallel mediation analysis to determine the mediating effect of decent work dimensions on the relationship between perceived physical activity enjoyment and perceived physical activity participation.

Effects	Unstandardised beta coefficients		Significance of beta coefficients			95% confidence interval	
	Beta	s.e.	*t*-value	*z*-value	*p*	LLCI	ULCI
**Direct effect(s)**							
**Fixed effects (within-effects specified)**							
*1. PAE (X) → PE (M*_1_ *)*	0.2689†	0.048	5.5819	-	< 0.0001	0.1744	0.3635
*2. PAE (X) → FE (M*_2_ *)*	0.4009†	0.056	7.1167	-	< 0.0001	0.2903	0.5114
*3. PAE (X) → TE (M*_3_ *)*	0.3652†	0.056	6.5016	-	< 0.0001	0.2549	0.4754
*4. PE (M*_1_ *) → PAP (Y)*	0.0331	0.018	1.7602	-	0.0787	−0.0038	0.0700
*5. FE (M*_2_ *) → PAP (Y)*	0.0393†	0.017	2.2720	-	0.0233	0.0054	0.0733
*6. TE (M*_3_ *) → PAP (Y)*	0.0343†	0.016	2.0247	-	0.0432	0.0010	0.0675
*7. PAE (X) → PAP (Y)*	0.0813†	0.024	3.3885	-	0.0007	0.0342	0.1283
**Indirect effect(s)**							
**Within – Indirect effects**							
*PE (M*_1_ *) as a mediator*	0.0089	0.005	-	1.6547	0.0980	−0.0009	0.0202
*FE (M*_2_ *) as a mediator*	0.0158†	0.007	-	2.1452	0.0319	0.0017	0.0311
*TE (M*_3_ *) as a mediator*	0.0125†	0.006	-	1.9926	0.0498	0.0010	0.0269

s.e., standard error; *X*, predictor/independent variable; *Y*, outcome/dependent variable; *PAE*, physical activity enjoyment; *PAP*, physical activity participation; *PE*, peer encouragement; *FE*, family encouragement; *TE*, teacher encouragement; *M*_1_, mediator variable 1; *M*_2_, mediator variable 2; *M*_3_, mediator variable 3.

† , Significant effect at alpha = 0.05.

## Discussion

Our aim was to assess the moderating role of social support factors on the relationship between physical activity enjoyment and physical activity participation levels among primary school children. This is important because it helps develop effective intervention programmes that promote physical activity participation. We provided tangible evidence of the influence of social support for both girls and boys in promoting PA participation. According to our results, peer, family and teacher encouragement were partial mediators in the relationship between perceived physical activity enjoyment and perceived physical activity participation. This is in support of other studies, which have shown that social support has a significant influence in promoting school children’s PA participation levels (Davison 2004). Further, the benefits of social support are also substantiated by several studies repeatedly showing that peer, family and teacher support are associated with higher levels of PA participation among children and adolescents (Haidar et al. 2019; Korcz et al. 2018).

The mediating role of peer support and/or encouragement makes sense from a developmental perspective, as peer support in young children is generally a dominant social factor that plays a critical role in developing PA motivation and engagement (Zhang et al. 2012). Our results are similar to the study by Smith (1999) in the sub-Saharan Africa. Another possible cause for the established mediating effect may be how children distance themselves from their parents and spend more time with peers or hang around with friends (Bortoli et al. 2018; Vitali et al. 2019). Peer support has been reported as having the same impact as parental support in promoting PA participation; however, peer support exceeds parental support for ages between 16 years and 18 years (Bokhorst et al. 2010). Thus, research suggests that peers have more influence on adolescent development in PA participation (Van Der Horst et al. 2007).

The significance of family encouragement as a partial mediator on the relationship between perceived PA enjoyment and perceived PA participation can be attributed to the form of support from parents that enables children to participate in sporting activities like buying equipment or payment of fees and transportation (Duncan et al. 2005). Thus, they will be promoting lifestyle behaviour (Dowda et al. 2007). In addition, families teach children skills and beliefs and act as role models, and this can assist in shaping important attitudes and behaviours associated with increasing PA participation in children (Edwardson et al. 2013). In our study, family encouragement was reported as having the most considerable mediating effect, followed by teacher encouragement, which may make sense because support from family is broad. There are siblings and parents in the family so family support can manifest in many different ways. This result is consistent with other studies that highlighted family encouragement as having a more significant impact in promoting PA participation among children (Duncan et al. 2005; Tremblay et al. 2015). It is also important to note that a lack of family encouragement decreases PA participation at the adolescent stage (Campos et al. 2019).

In our study, teacher encouragement was also found to be partially mediating the relationship between perceived PA enjoyment and perceived PA participation. It is second after the family encouragement, according to our findings. The teacher’s influence starts in the classroom when teaching life orientation (or physical education) and extra-curricular sports in the school. The learners will then apply the skills taught in class and in the extra-curricular activities to participate in physical activities to improve their health throughout their lives. Several studies have also shown that teachers can instill children’s intrinsic motivation for PA and their perceived sporting games competence when they support PA goals and provide positive feedback in a classroom environment (Koka & Hein 2003). Furthermore, teachers have an influential role in learning in the general classroom environment, where children imitate the teacher’s knowledge, role modelling, behaviours and opinions. Our results are similar to other studies, which found that teacher support was more effective in small children but lower in older age groups, and this may have been caused by the changes that take place from lower grades to upper grades. The same study also showed that girls alone receive more support from teachers than boys (Bokhorst et al. 2010).

Our results on gender indicated that boys participate in PA more than girls. These findings contradict the results in Europe, whereby more than two-thirds of European children are active regardless of gender differences (Zembura et al. 2018). However, as stated by Dumith et al. (2011), although PA participation decline among girls was greater between 9 and 12 years old, among boys, it was greater at the ages of 13 and 16 years. The differences in PA participation between boys and girls may be caused by differences in social support provided by parents and peers (Cheng et al. 2020). In terms of place of residence, our study supports the findings of Gomwe et al. (2022) that rural children participate more in PA as compared to urban and peri-urban children. This may be attributed to the differences in lifestyle within environment settings. Most children walk long distances to and from school in rural areas and engage in more physical household chores than in urban and peri-urban settings (Gomwe et al. 2022). The urban environment could be less conducive to PA participation for safety reasons. The high crime rate in urban neighbourhood decreases PA participation levels.

### Limitations of the study

Potential sources of bias were of great concern in our study. The major form of bias was selection bias. Thus, in our study, the sampling method and availability of potential participants may have affected the extent of selection bias. Our study was population-based. Hence, the resultant sample is likely to represent the population, thereby minimising selection bias. A random sampling method was used on the population, and selection bias was minimised further.

The limitation of our study is that the questionnaire was used to gather information about the learners’ participation in a broad spectrum of different physical activities across several domains. Minor errors may have occurred because of the age of the learners. However, the purpose of using the Physical Activity Questionnaire for Older Children (PAQ-C), previously used and validated in an ethnically diverse and similar cohort of South African school learners, was to obtain a general idea of their physical activity participation.

## Conclusion

There were low levels of perceived physical activity participation among the school learners. Peer, family and teacher encouragement partially mediates the relationship between perceived PA enjoyment and perceived PA participation. Our study has shown that family encouragement has the largest mediating effect, followed by teacher encouragement. To prevent further low levels of perceived PA participation among school learners, a supportive social environment around PA participation must be implemented, focusing more on enhancing the roles of family and teachers in supporting PA participation in children.

## References

[CIT0001] Bandura, A., 1986, *Social foundations of thought and action*, pp. 23–28, Prentice–Hall, Englewood Cliffs, New Jersey.

[CIT0002] Bandura, A. & Walters, R.H., 1977, *Social learning theory*, vol. 1, Prentice Hall, Englewood Cliffs, New Jersey.

[CIT0003] Beets, M.W., Pitetti, K.H. & Forlaw, L., 2007, ‘The role of self-efficacy and referent specific social support in promoting rural adolescent girls’ physical activity’, *American Journal of Health Behavior* 31(3), 227–237. 10.5993/AJHB.31.3.1 17402863

[CIT0004] Biddle, S. & Goudas, M., 1996, ‘Analysis of children’s physical activity and its association with adult encouragement and social cognitive variables’, *Journal of School Health* 66(2), 75–78. 10.1111/j.1746-1561.1996.tb07914.x 8930014

[CIT0005] Birnbaum, A.S., Evenson, K.R., Motl, R.W., Dishman, R.K., Voorhees, C.C., Sallis, J.F. et al., 2005, ‘Scale development for perceived school climate for girls’ physical activity’, *American Journal of Health Behavior* 29(3), 250–257. 10.5993/AJHB.29.3.6 15899688 PMC2494732

[CIT0006] Bokhorst, C.L., Sumter, S.R. & Westenberg, P.M., 2010, ‘Social support from parents, friends, classmates, and teachers in children and adolescents aged 9 to 18 years: Who is perceived as most supportive?’, *Social Development* 19(2), 417–426. 10.1111/j.1467-9507.2009.00540.x

[CIT0007] Bortoli, L., Vitali, F., Di Battista, R., Ruiz, M.C. & Robazza, C., 2018, ‘Initial validation of the Psychobiosocial States in Physical Education (PBS-SPE) scale’, *Frontiers in Psychology* 9, 2446. 10.3389/fpsyg.2018.02446 30574110 PMC6291472

[CIT0008] Bronikowski, M., Bronikowska, M., Maciaszek, J. & Glapa, A., 2018, ‘Maybe it is not a goal that matters-a report from a physical activity intervention in youth’, *Journal of Sports Medicine and Physical Fitness* 58(3), 348–355. 10.23736/S0022-4707.16.06611-1 29473720

[CIT0009] Bull, F.C., Al-Ansari, S.S., Biddle, S., Borodulin, K., Buman, M.P., Cardon, G. et al., 2020, ‘World Health Organization 2020 guidelines on physical activity and sedentary behaviour’, *British Journal of Sports Medicine* 54(24), 1451–1462. 10.1136/bjsports-2020-102955 33239350 PMC7719906

[CIT0010] Campos, J.G., Bacil, E.D.A., Piola, T.S., Silva, M.P.D., Pacífico, A.B. & Campos, W.D., 2019, ‘Social support, self-efficacy and level of physical activity of students aged 13–15 years’, *Revista Brasileira de Cineantropometria & Desempenho Humano* 21, e58684. 10.1590/1980-0037.2019v21e58684

[CIT0011] Cheng, L.A., Mendonça, G., Lucena, J., Rech, C.R. & Farias Júnior, J.C., 2020, ‘Is the association between sociodemographic variables and physical activity levels in adolescents mediated by social support and self-efficacy?’, *Jornal de Pediatria* 96(1), 46–52. 10.1016/j.jped.2018.08.003 30236591 PMC9432328

[CIT0012] Coetzee, D., Du Toit, D. & De Vos, J.C., 2016, ‘The types and levels of physical activity and sedentary behaviour of Senior Phase learners in Potchefstroom’, *Health SA Gesondheid* 21(1), 372–380. 10.4102/hsag.v21i0.994

[CIT0013] Crocker, P.R., Bailey, D.A., Faulkner, R.A., Kowalski, K.C. & McGrath, R., 1997, ‘Measuring general levels of physical activity: Preliminary evidence for the Physical Activity Questionnaire for Older Children’, *Medicine and Science in Sports and Exercise* 29(10), 1344–1349. 10.1097/00005768-199710000-00011 9346166

[CIT0014] Crocker, P.R., Bouffard, M. & Gessaroli, M.E., 1995, ‘Measuring enjoyment in youth sport settings: A confirmatory factor analysis of the Physical Activity Enjoyment Scale’, *Journal of Sport and Exercise Psychology* 17(2), 200–205. 10.1123/jsep.17.2.200

[CIT0015] Davison, K.K., 2004, ‘Activity-related support from parents, peers, and siblings and adolescents’ physical activity: Are there sex differences?’, *Journal of Physical Activity and Health* 1(4), 363–376. 10.1123/jpah.1.4.363

[CIT0016] Dowda, M., Dishman, R.K., Pfeiffer, K.A. & Pate, R.R., 2007, ‘Family support for physical activity in girls from 8th to 12th grade in South Carolina’, *Preventive Medicine* 44(2), 153–159. 10.1016/j.ypmed.2006.10.001 17157371 PMC2031210

[CIT0017] Dumith, S.C., Gigante, D.P., Domingues, M.R. & Kohl, III, H.W., 2011, ‘Physical activity change during adolescence: A systematic review and a pooled analysis’, *International Journal of Epidemiology* 40(3), 685–698. 10.1093/ije/dyq272 21245072

[CIT0018] Duncan, S.C., Duncan, T.E. & Strycker, L.A., 2005, ‘Sources and types of social support in youth physical activity’, *Health Psychology* 24(1), 3. 10.1037/0278-6133.24.1.3 15631557

[CIT0019] Eather, N., Morgan, P.J. & Lubans, D.R., 2013, ‘Feasibility and preliminary efficacy of the Fit4Fun intervention for improving physical fitness in a sample of primary school children: A pilot study’, *Physical Education and Sport Pedagogy* 18(4), 389–411. 10.1080/17408989.2012.690375

[CIT0020] Edwardson, C.L., Gorely, T., Pearson, N. & Atkin, A., 2013, ‘Sources of activity-related social support and adolescents’ objectively measured after-school and weekend physical activity: Sex and age differences’, *Journal of Physical Activity and Health* 10(8), 1153–1158. 10.1123/jpah.10.8.1153 23223792

[CIT0021] Estevan, I., Menescardi, C., García-Massó, X., Barnett, L.M. & Molina-García, J., 2021, ‘Profiling children longitudinally: A three-year follow-up study of perceived and actual motor competence and physical fitness’, *Scandinavian Journal of Medicine & Science in Sports* 31(s1), 35–46. 10.1111/sms.13731 33871084

[CIT0022] Fairclough, S., Stratton, G. & Baldwin, G., 2002, ‘The contribution of secondary school physical education to lifetime physical activity’, *European Physical Education Review* 8(1), 69–84. 10.1177/1356336X020081005

[CIT0023] Gomwe, H., Seekoe, E., Lyoka, P., Marange, C.S. & Mafa, D., 2022, ‘Physical activity and sedentary behaviour of primary school learners in the Eastern Cape province of South Africa’, *South African Family Practice* 64(2), e1–e8. 10.4102/safp.v64i1.5381 PMC8991265 35384676

[CIT0024] Haidar, A., Ranjit, N., Archer, N. & Hoelscher, D.M., 2019, ‘Parental and peer social support is associated with healthier physical activity behaviors in adolescents: A cross-sectional analysis of Texas School Physical Activity and Nutrition (TX SPAN) data’, *BMC Public Health* 19(1), 1–9. 10.1186/s12889-019-7001-0 31132999 PMC6537420

[CIT0025] Hayes, A.F. & Rockwood, N.J., 2020, ‘Conditional process analysis: Concepts, computation, and advances in the modeling of the contingencies of mechanisms’, *American Behavioral Scientist* 64(1), 19–54. 10.1177/0002764219859633

[CIT0026] Johnson, C.C., Li, D., Epping, J., Lytle, L.A., Cribb, P.W., Williston, B.J. et al., 2000, ‘A transactional model of social support, self-efficacy, and physical activity of children in the child and adolescent trial for cardiovascular health’, *Journal of Health Education* 31(1), 2–9. 10.1080/10556699.2000.10608640

[CIT0027] Kendzierski, D. & DeCarlo, K.J., 1991, ‘Physical activity enjoyment scale: Two validation studies’, *Journal of Sport & Exercise Psychology* 13(1), 50–64. 10.1123/jsep.13.1.50

[CIT0028] Kohl, III, H.W. & Cook, H.D. (eds.), 2013, *Educating the student body: Taking physical activity and physical education to school*, National Academies Press (US), Washington, DC. 24851299

[CIT0029] Koka, A. & Hein, V., 2003, ‘Perceptions of teacher’s feedback and learning environment as predictors of intrinsic motivation in physical education’, *Psychology of Sport and Exercise* 4(4), 333–346. 10.1016/S1469-0292(02)00012-2

[CIT0030] Korcz, A., Bronikowska, M., Laudanska-Krzeminska, I., Borowiec, J., Ludwiczak, M. & Bronikowski, M., 2018, ‘Is social support during physical education lessons associated with body mass index status, sex and age?’, *South African Journal for Research in Sport, Physical Education and Recreation* 40(2), 53–68.

[CIT0031] Kowalski, K.C., Crocker, P.R. & Faulkner, R.A., 1997, ‘Validation of the physical activity questionnaire for older children’, *Pediatric Exercise Science* 9(2), 174–186.

[CIT0032] Kowalski, K.C., Crocker, P.R. & Donen, R.M., 2004, ‘The physical activity questionnaire for older children (PAQ-C) and adolescents (PAQ-A) manual’, *College of Kinesiology, University of Saskatchewan* 87(1), 1–38.

[CIT0033] Lubans, D.R., Foster, C. & Biddle, S.J., 2008, ‘A review of mediators of behavior in interventions to promote physical activity among children and adolescents’, *Preventive Medicine* 47(5), 463–470. 10.1016/j.ypmed.2008.07.011 18708086

[CIT0034] MacKinnon, D.P., Fairchild, A.J. & Fritz, M.S., 2007, ‘Mediation analysis’, *Annual Review of Psychology* 58, 593–614. 10.1146/annurev.psych.58.110405.085542 PMC2819368 16968208

[CIT0035] McMinn, A.M., Van Sluijs, E.M., Wedderkopp, N., Froberg, K. & Griffin, S.J., 2008, ‘Sociocultural correlates of physical activity in children and adolescents: Findings from the Danish arm of the European Youth Heart study’, *Pediatric Exercise Science* 20(3), 319–332. 10.1123/pes.20.3.319 18714121

[CIT0036] McVeigh, J.A. & Norris, S.A., 2012, ‘Criterion validity and test-retest reliability of a physical activity questionnaire in South African primary school-aged children’, *South African Journal of Sports Medicine* 24(2), 43–48.

[CIT0037] Menescardi, C. & Estevan, I., 2021, ‘Parental and peer support matters: A broad umbrella of the role of perceived social support in the association between children’s perceived motor competence and physical activity’, *International Journal of Environmental Research and Public Health* 18(12), 6646. 10.3390/ijerph18126646 34205557 PMC8296426

[CIT0038] Michie, S. & Abraham, C., 2004, ‘Interventions to change health behaviours: Evidence-based or evidence-inspired?’, *Psychology & Health* 19(1), 29–49. 10.1080/0887044031000141199

[CIT0039] Monteiro, D., Rodrigues, F. & Lopes, V.P., 2021, ‘Social support provided by the best friend and vigorous-intensity physical activity in the relationship between perceived benefits and global self-worth of adolescents’, *Revista de Psicodidáctica* 26(1), 70–77. 10.1016/j.psicod.2020.11.004

[CIT0040] Moore, J.B., Hanes, J.C., Barbeau, P., Gutin, B., Treviño, R.P. & Yin, Z., 2007, ‘Validation of the Physical Activity Questionnaire for Older Children in children of different races’, *Pediatric Exercise Science* 19(1), 6–19. 10.1123/pes.19.1.6 17554153

[CIT0041] Morrissey, J.L., Janz, K.F., Letuchy, E.M., Francis, S.L. & Levy, S.M., 2015, ‘The effect of family and friend support on physical activity through adolescence: A longitudinal study’, *International Journal of Behavioral Nutrition and Physical Activity* 12, 1–9. 10.1186/s12966-015-0265-6 26289232 PMC4545918

[CIT0042] Prochaska, J.J., Rodgers, M.W. & Sallis, J.F., 2002, ‘Association of parent and peer support with adolescent physical activity’, *Research Quarterly for Exercise and Sport* 73(2), 206–210. 10.1080/02701367.2002.10609010 12092896

[CIT0043] Sallis, J.F., McKenzie, T.L., Alcaraz, J.E., Kolody, B., Faucette, N. & Hovell, M.F., 1997, ‘The effects of a 2-year physical education program (SPARK) on physical activity and fitness in elementary school students. Sports, play and active recreation for kids’, *American Journal of Public Health* 87(8), 1328–1334. 10.2105/AJPH.87.8.1328 9279269 PMC1381094

[CIT0044] Sallis, J.F., Prochaska, J.J. & Taylor, W.C., 2000, ‘A review of correlates of physical activity of children and adolescents’, *Medicine and Science in Sports and Exercise* 32(5), 963–975. 10.1097/00005768-200005000-00014 10795788

[CIT0045] Salmon, J., Brown, H. & Hume, C., 2009, ‘Effects of strategies to promote children’s physical activity on potential mediators’, *International Journal of Obesity* 33(1), S66–S73. 10.1038/ijo.2009.21 19363512

[CIT0046] Saunders, R.P., Pate, R.R., Felton, G., Dowda, M., Weinrich, M.C., Ward, D.S., et al., 1997, ‘Development of questionnaires to measure psychosocial influences on children’s physical activity’, *Preventive Medicine* 26(2), 241–247. 9085394 10.1006/pmed.1996.0134

[CIT0047] Smith, A.L., 1999, ‘Perceptions of peer relationships and physical activity participation in early adolescence’, *Journal of Sport & Exercise Psychology* 21(4), 329–350. 10.1123/jsep.21.4.329

[CIT0048] Tremblay, M.S., Gray, C., Babcock, S., Barnes, J., Costas Bradstreet, C., Carr, D. et al., 2015, ‘Position statement on active outdoor play’, *International Journal of Environmental Research and Public Health* 12(6), 6475–6505. 26062040 10.3390/ijerph120606475 PMC4483712

[CIT0049] Van Biljon, A., McKune, A.J., DuBose, K.D., Kolanisi, U. & Semple, S.J., 2018, ‘Physical activity levels in urban-based South African learners: A cross-sectional study of 7 348 participants’, *South African Medical Journal* 108(2), 126–131. 10.7196/SAMJ.2018.v108i2.12766 29429445

[CIT0050] Van Der Horst, K., Paw, M.J.C.A., Twisk, J.W. & Van Mechelen, W., 2007, ‘A brief review on correlates of physical activity and sedentariness in youth’, *Medicine & Science in Sports & Exercise* 39(8), 1241–1250. 10.1249/mss.0b013e318059bf35 17762356

[CIT0051] Vitali, F., Robazza, C., Bortoli, L., Bertinato, L., Schena, F. & Lanza, M., 2019, ‘Enhancing fitness, enjoyment, and physical self-efficacy in primary school children: A DEDIPAC naturalistic study’, *PeerJ* 7, e6436. 10.7717/peerj.6436 30809444 PMC6387574

[CIT0052] WHO, 2010, *Global recommendations on physical activity for health*, World Health Organization, Geneva. 26180873

[CIT0053] Zembura, P., Korcz, A., Cieśla, E., Gołdys, A. & Nałȩcz, H., 2018, ‘Results from Poland’s 2018 report card on physical activity for children and youth’, *Journal of Physical Activity and Health* 15(s2), S395–S397. 10.1123/jpah.2018-0540 30475115

[CIT0054] Zhang, T., Solmon, M.A., Gao, Z. & Kosma, M., 2012, ‘Promoting school students’ physical activity: A social ecological perspective’, *Journal of Applied Sport Psychology* 24(1), 92–105. 10.1080/10413200.2011.627083

